# Diagnostic value of DNA alteration: loss of heterozygosity or allelic imbalance—promising for molecular staging of prostate cancers

**DOI:** 10.1007/s12032-012-0391-9

**Published:** 2013-01-04

**Authors:** Magdalena Bryś, Monika Migdalska-Sęk, Dorota Pastuszak-Lewandoska, Ewa Forma, Karolina Czarnecka, Daria Domańska, Ewa Nawrot, Jacek Wilkosz, Waldemar Różański, Ewa Brzeziańska

**Affiliations:** 1 Department of Cytobiochemistry, Faculty of Biology and Environmental Protection, University of Łódź, Pomorska St. 141/143, 90-236 Lodz, Poland; 2 Department of Molecular Bases of Medicine, Medical University of Łódź, Pomorska St. 251, 92-213 Lodz, Poland; 3 2nd Department of Urology, Medical University of Łódź, Pabianicka 62, 93-513 Lodz, Poland

**Keywords:** Prostate cancer, LOH, MSI, Molecular markers, TRUS-guided prostate biopsy

## Abstract

The biological behavior of prostate cancer is uncertain, and therefore, search for molecular prognostic markers associated with disease progression seems to be essential. We performed microsatellite allelotyping of DNA isolated from primary prostate tumors biopsies (prostate adenocarcinoma, PCa). We evaluated the frequency of allelic imbalance (AI), including loss of heterozygosity and/or microsatellite imbalance (LOH/MSI) as well as the association of these DNA alterations with clinicopathological variables. We assessed the significance of LOH/MSI alterations in selected imprinted and non-imprinted chromosomal regions (IR and NIR) in PCa. A total of 50 biopsies of prostate tumor (containing >75 % tumor cells) were histologically examined confirming prostate carcinoma. Microsatellite allelotyping using 16 microsatellite markers linked to the following chromosomal regions: 1p31.2, 3p21.3–25.3, 7q32.2, 9p21.3, 11p15.5, 12q23.2, and 16q22.1 was performed. The incidence of LOH/MSI alterations in prostate tumor cells was the highest for chromosomal regions 7q32.2 and 16q22.1 (31.25 and 26.60 %, respectively), followed by 1p31.2 and 3p21.3–25.3 (26.50 and 17.40 %, respectively). Statistically significant increase in LOH/MSI alterations has been observed for markers: D1S2137 (1p region; *p* = 0.00032), D9S974 (9p region; *p* = 0.0017), and D16S3025 (16q region; *p* = 0.0017). Statistically significant differences in frequency of LOH/MSI alterations in particular chromosomal regions have been found for 1p31.2, 7q32.2 and 16q22.1 (*p* = 0.027, *p* = 0.012 and *p* = 0.031, respectively). We documented statistically significant association between Fractional Allele Loss (FAL) index and advanced tumor stage (*p* < 0.05). We suggest that genomic instability of LOH/MSI type is a frequent event in prostate carcinogenesis and assessed as FAL index has clinical value for the molecular staging of prostate cancer in (TRUS)-guided prostate biopsy material.

## Introduction

Prostate cancer is one of the most common non-skin cancer affecting older men (>65 years) worldwide, and among US men, it is the second leading cause of cancer death [1]. However, the incidence of this type of cancer varies widely depending on population [2]. Prostate adenocarcinoma, developing in glandular tissue, represents a significant event in prostate carcinogenesis, accounting for 95 % of all prostate cancers [3]. This malignancy starts in the prostate gland and, if not treated successfully at an early stage, in approximately 65–75 % cases spread in short term to other parts of the body, where mainly bone metastases are observed [4]. The etiology of this type of tumor is complex and poorly understood, but genetic component is widely documented, not only in case of hereditary prostate cancer, but also in sporadic cases. The molecular mechanisms leading to the development of prostate cancer and to the progression of the disease are still under the consideration. It is estimated that as much as 42 % of the risk of prostate cancer may be attributed to genetic influences, including individual predisposition, highly penetrant genes, more commonly weakly penetrant genes, and gene–environment interactions. Several advanced studies focused on molecular genetics of epithelial cancers and their progression—including prostate cancer—suggested the involvement of numerous oncogenes in their carcinogenesis via activated somatic mutations, loss of tumor suppressor gene functions (in particular *Tp53*), as well as mutator gene defects (mismatch repair gene mutations, MMR) [5]. As a result, prostate cancer susceptibility *loci* have been reported [6].

Recently, chromosomal instability involving alterations in chromosomal segregation and/or structure via deletion, duplication, as well as microsatellite instability (MSI) and/or loss of heterozygosity (LOH) has been described as a distinct type of genetic instability, characteristic for prostate cancer [7, 8]. Many studies based on a large number of microsatellite markers unequivocally confirmed the presence of high level of allelic imbalances in prostate cancer [7]. Moreover, as a final result of these observations, hypothetic model for genetic pathways in prostatic carcinogenesis was proposed [8]. It should be stressed that especially MSI was recognized as distinct pattern of molecular alterations in prostate tumors, not related to MMR defects, *Tp53* alterations, or histopathological characteristics [9]. However, there are some controversial reports, where allelic imbalance (mainly MSI) seems to play a trivial role in prostate carcinogenesis, even in advanced prostate cancer [9].

The aim of our study was to answer the question whether LOH/MSI represents the important mechanism in prostate tumorigenesis. Our additional goal was to identify molecular markers associated with disease progression. In order to investigate whether an allelic imbalance (AI) is associated with clinicopathological variables of patients with prostate cancer, we investigated the incidence of LOH and MSI in sporadic adenocarcinomas in different stage of clinical progression, focusing on selected *loci* including suppressor genes located in imprinting regions (IRs) and non-Imprinting regions (NIRs) of human genome. Finally, we evaluated the clinical value of LOH/MSI incidence for the molecular staging of prostate cancer.

## Materials and methods

### Specimens

The studied biological material was received from 2nd Department of Urology, Medical University of Lodz, Poland, between October 2009 and December 2011 and comprised of 50 prostate cancer biopsy specimens. All samples were obtained from the peripheral zone of prostate gland in patients who underwent transrectal ultrasound (TRUS)-guided prostate biopsy. All tissues were selected and evaluated by the independent pathologists who determined Gleason grading and differentiation status. None of the recruited patients received preoperative chemo- or radiotherapy. The pathological evaluation report was obtained for each patient. Clinical and demographic characteristics of study subjects are presented in Table 1.

**Table 1:** Demographic and clinical characteristics of study subjects

Age at biopsy (year)	Median 71 (range 55–86)
≤70	22 (44 %)
>70	28 (56 %)
Total PSA (TPSA, ng/ml)^a^	
≥0–10	8 (16 %)
≥10–100	35 (70 %)
>100	7 (14 %)
Free/total PSA (F/T PSA)^a^	
≤0.12	13 (26 %)
0.13–0.16	14 (28 %)
>0.16	23 (46 %)
PSA density (PSAD, ng/ml)^a^	
≤0.15	12 (24 %)
>0.15	38 (76 %)
Prostate volume (ml)	
<30	16 (32 %)
30–50	21 (42 %)
≥50	13 (26 %)
DRE (digital rectal examination)	
0	19 (38 %)
1	31 (62 %)
TRUS (transrectal ultrasound)	
0	14 (28 %)
1	36 (72 %)
Gleason score, biopsy based	
>7	29 (58 %)
≤7	21 (42 %)
American joint commission on cancer staging T stage	
T1c	12 (24 %)
T2b	2 (4 %)
T2c	12 (24 %)
T3a	5 (10 %)
T3b	7 (14 %)
T4	12 (24 %)

^a^PSA as measured at the time of diagnosis

Immediately after (TRUS)-guided prostate biopsy, the obtained samples were placed in RNAlater^®^ solution (Qiagen, Inc., Chatsworth, CA, USA) and stored at −70 °C. Simultaneously, blood samples (2 ml) from each patient were collected on EDTA and frozen. Informed consent was obtained from patients, and this study was approved by the Ethical Committee of the Medical University of Lodz (RNN/59/09/KE).

### DNA isolation

Isolation of genomic DNA from prostatic biopsies and blood samples—which served as a reference DNA—was performed using QIAamp DNA Investigator Kit (Qiagen, Germany), according to the manufacturer’s protocol. Quality and quantity of each DNA sample was spectrophotometrically assessed, measuring absorbance at wave length of 260/280 nm (Eppendorf BioPhotometr™ Plus, Eppendorf, Germany). DNA with a 260/280 nm ratio in range 1.8–2.0 was considered as high quality.

### Microsatellite analysis

Sixteen primer pairs were used to perform microsatellite analysis, based on amplification of polymorphic microsatellite repeats: (T)n, (CA)n, (TTA)n, and (TCTA)n in paired DNA samples, i.e., obtained from prostatic tumor cells and blood (control sample, reference DNA) from the same patient. These markers linked to the following chromosomal regions: 1p31.2, 3p21.3–25.3, 7q32.2, 9p21.3, 11p15.5, 12q23.2, and 16q22.1. In LOH/MSI analysis, 9 microsatellite markers mapped to known IRs, and 7 to NIRs of human genome, were used. Nucleotide sequences of microsatellite markers used in the study and their chromosomal localization are presented in Tables 2 and 3. The choice of markers was compatible to the loci of important genes involved in important cell processes during carcinogenesis. The selected genes from these regions are pivotal for cell cycle regulation, proliferation, and adhesion. All primer sequences of the used markers and their cytogenetic localizations were found in NCBI database (http://www.ncbi.nlm.nih.gov/genome/sts/sts) with supplementary mapping information, if necessary, provided in Cooperative Human Linkage Centre Database (http://www.chlc.org), the Genome Database (http://www.gdb.org). Each forward primer was labeled at 3′end with fluorescent dye: 6-FAM, NED, PET, or VIC.

**Table 2:** Nucleotide sequences of microsatellite markers and their chromosomal localization from the imprinted region (IR) of the genome

Chromosomal localization region	Nucleotide sequence of microsatellite marker (5′–3′)	
	Sense	Antisense
1p31.2		
D1S2137	ACATCTTTGGTTTGGATAGATG	CAAAACTGCACATTTTGCAC
D1S368	GGGCATTGTTTAGGGGTG	TAGTGGGCTTTACGTCTGC
7q32.2		
D7S2519	GGAGGTTAAGATTTACAG	GCTGTGGTGTATCCTGTG
D7S2544	TCCCCAGACCCCATTC	TCCTGTTCATCCTTCATTCC
D7S530	CGTTGCATTTTAGTGGAGCACAG	CAGCAGTAATGAAAGCAAAACACAG
9p21.3		
D9S974	GAGCCTGGTCTGGATCATAA	AAGCTTACAGAACCAGACAG
D9S1604	CCTGGGTCTCCAATTTGTCA	AGCACATGACACTGTGTGTG
11p15.5		
D11S4088	GGGCAGAGGCAGTGGAG	GCATGTTTCGGGGGTG
D11S1318	CCCGTATGGCAACAGG	TGTGCATGTNCATGAGTG

**Table 3:** Nucleotide sequences of microsatellite markers and their chromosomal localization from the non-imprinted region (NIR) of the genome

Chromosomal localization region	Nucleotide sequence of microsatellite marker (5′–3′)	
	Sense	Antisense
3p21.3		
D3S3615	TGGAAAGGTAAGCACAAGC	TCCTCCCAGGAAGCAC
3p25.3		
D3S1317	TACAAGTTCAGTGGAGAACC	CCTCCAGGCCATACACAGTCA
D3S3611	GCTACCTCTGCTGAGCAT	TAGCAAGACTGTTGGGG
12q23.2		
D12S1041	AACTGTGGAAAAAGGGGAAC	TGCAACAAACCACCATGG
D12S1727	AGTCACCACTGAAAATCCAC	GAGTGAGACCCCGTAAAAA
16q22.1		
D16S496	GAAAGGCTACTTCATAGATGGCAAT	ATAAGCCACTGCGCCCAT
D16S3025	TCCATTGGACTTATAACCATG	AGCTGAGAGACATCTGGG

Amplification reactions with microsatellite markers were performed in a Personal Thermocycler (Eppendorf, Germany), in a total volume of 25 μl, including 10× AmpliTaq Gold^®^ 360 buffer (Applied Biosystems, USA), 360 GC Enhancer (Applied Biosystems, USA), 5 U/μl AmpliTaq Gold^®^ 360 DNA Polymerase (Applied Biosystems, USA), 25 mM MgCl_2_, 10 mM dNTPs (Applied Biosystems, USA), 30–40 ng DNA, 0.5 μM each primer and nuclease-free water. Each microsatellite marker was amplified at its own specific annealing temperature to optimalize the PCRs.

PCR conditions were as follows: initial denaturation at 95 °C for 10 min, then 30 cycles of amplification with denaturation at 95 °C for 45 s, primer annealing for 30 s at temperature specific for each marker, i.e., in the range of 47–50 °C (for D3S3615, D3S1317, D3S3611, D7S2519, D7S2544, D11S4088, D11S1318, D12S1041, D12S1727, D16S3025), 51–59 °C (for D1S2137, D1S368, D7S530, D9S974, D9S1604, D16S496), followed by elongation step at 72 °C for 1 min.

Afterward, PCR product (0.5 μl) was mixed with 0.25 μl GS500-LIZ Size Standard (Applied Biosystems, USA) and Hi-Di™ Formamide (Applied Biosystems, USA) up to the final volume of 10 μl. The obtained mixture was denatured for 5 min at 95 °C, then cooled on ice for 3 min, and separated by capillary electrophoresis in 3130xl Genetic Analyzer (Applied Biosystems, Hitachi, USA) using GeneMapper Software v 4.0, according to the manufacturer’s protocol. Study samples were considered informative, i.e., heterozygous, when two distinct alleles were visible in the reference leukocyte DNA sample (from blood of each patient). Evaluation of LOH/MSI was performed by calculating the ratio of the fluorescence intensity of the alleles, originating from blood sample (N, normal, i.e., control sample) to the fluorescence intensity of the alleles originating from prostatic biopsy (T, tumor). For each informative tumor–normal DNA pair (paired T and N samples), an Allelic Imbalance Ratio (AIR) was calculated, based on the maximum allele peak heights (fluorescence intensity), as follows: normal allele 1: normal allele 2/tumor allele 1: tumor allele 2 (N1:N2/T1:T2) according to protocol [10]. When AIR was less than 0.67 or greater than 1.35, it was considered indicative of LOH in tumor samples (according to the criteria of GeneMapper Software v 4.0). Tumor DNA was considered as harboring MSI if one or more additional alleles were present in tumor DNA sample, as compared with the control DNA sample. For each prostate adenocarcinoma sample, Fractional Allele Loss (FAL) index was also calculated reflecting the ratio of total number of chromosomal *loci* with LOH or MSI to the total number of informative *loci* examined.

### Statistical analysis

Since values determined in experiments did not show normal distribution (Kolmogorov–Smirnov test), the nonparametrical statistical tests (Mann–Whitney *U* test, Kruskal–Wallis test) were applied. Statistical significance was determined at the level of *p* < 0.05. For calculations, Statistica for Windows v. 10 (StatSoft, Poland) program was applied.

## Results

Demographic and clinical characteristics of study subjects are presented in Table 1.

**Fig. 1:**
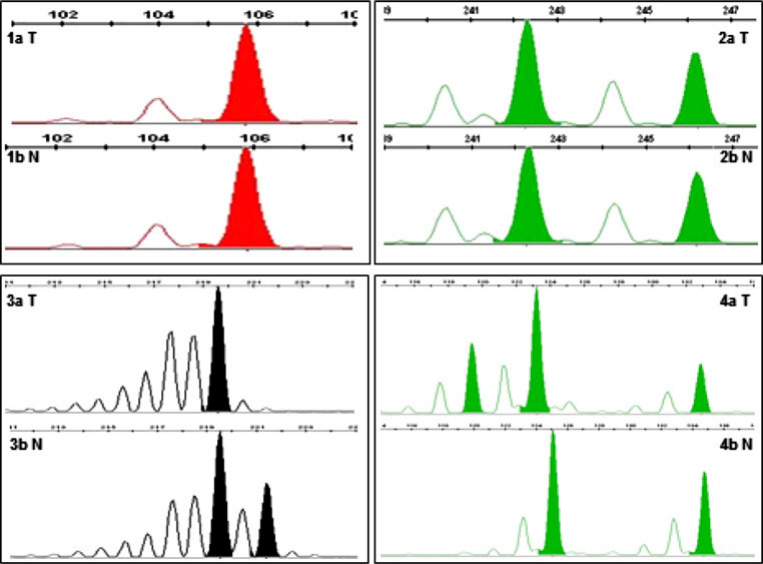
LOH/MSI analysis in prostate adenocarcinoma samples (3130xl Genetic Analyzer, GeneMapper Software v. 4.0; Applied Biosystems, Hitachi).* 1aT* homozygous DNA from patient with diagnosed prostate adenocarcinoma (sample no. 134; D16S3025 marker);* 1bN* homozygous DNA from blood sample from the same patient;* 2aT* heterozygous DNA from patient with diagnosed prostate adenocarcinoma (sample no. 21; D7S530 marker);* 2bN* heterozygous DNA from blood sample from the same patient;* 3aT* LOH in DNA from the patient with diagnosed prostate adenocarcinoma (sample no. 138; D16S496 marker);* 3bN* heterozygous DNA from blood sample from the same patient;* 4aT* MSI in DNA from the patient with diagnosed prostate adenocarcinoma (sample no. 138; D11S1318 marker);* 4bN* heterozygous DNA from blood sample from the same patient; *N* normal (blood sample), *T* the biopsy sample of prostate cancer

Using the panel of 16 microsatellite markers, LOH/MSI analysis was performed in DNA from all (50) patients. The normativity of the markers was assessed to be in the range of 52–88 % (mean 74 % ± 0.12). All the studied DNA (50 samples) derived from prostate adenocarcinoma biopsies were informative at least for two studied *loci*. LOH/MSI changes were observed for 15 out of 16 (93.75 %) microsatellite markers. The obtained results indicate that in all (50) studied prostate adenocarcinoma biopsies, LOH/MSI frequency was present in the range of 6.80–31.25 % (mean 20.18 % ± 0.08), depending on the chromosomal region. Representative examples of LOH/MSI in prostate adenocarcinoma cells are shown in Fig. 1.

We evaluated the frequency of LOH/MSI alteration for each marker used in the study, separately. The most frequent (21.21 %; 7/33 informative *loci*) LOH/MSI alteration has been observed for D16S3025 marker, spanning the chromosomal region 16q22.1, followed by D1S2137 marker (20.0 %; 8/40 informative *loci*), covering the chromosomal region 1p31.2. Additionally, for D16S496 marker—localized also at 16q22.1—the frequency of LOH in the studied DNA samples was 14.70 % (5/34 informative loci), and 12.50 % (5/40 informative loci) for D1S368 (1p31.2). Genetic instabilities of LOH/MSI type were also observed for D3S3615, D7S2544, D7S530, and D9S974 markers with similar frequencies, which were as follows: 13.20, 12.20, 15.80, and 15.90 %, respectively. The lowest incidence of LOH/MSI (<10 %) was observed for markers: D3S3611, D7S2519, D9S1604, D11S4088, D11S1318, D12S1041, and D12S1727. The LOH/MSI alteration has not been found for D3S1317 marker. Statistically significant increase in LOH/MSI alterations has been observed for markers: D1S2137 (1p region; *p* = 0.00032), D9S974 (9p region; *p* = 0.0017), and D16S3025 (16q region; *p* = 0.0017).

**Fig. 2:**
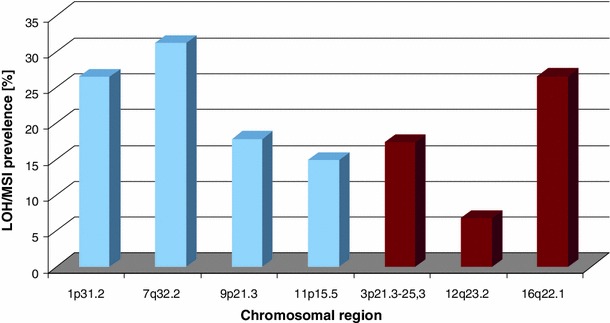
LOH/MSI frequency in prostate adenocarcinoma samples in the studied chromosomal region (i.e., 1p31.2, 7q32.2, 9p21.3, 11p15.5, 3p21.3–25.3, 16q22.1) as well as in relation to imprinted or non-imprinted regions (IR, NIR, respectively)

Focusing on a comparison of LOH/MSI total frequency between the studied chromosomal regions (for all markers used in each region), the highest frequency of LOH/MSI was observed at 7q32.2 (31.25 %), 16q22.1 (26.60 %), and 1p31.2 (26.50 %) and lower in 9p21.3 (17.80 %), 3p21.3–25.3 (17.40 %) and 11p15.5 (14.90 %). The lowest frequency of LOH/MSI was found at 12q23.2 (6.80 %) chromosomal region (see Fig. 2).

The total frequency of LOH/MSI was also referred to two different genomic regions under the study: IR and NIR (see Fig. 2). The corresponding microsatellite markers used in the study are listed in Tables 2 and 3. The most frequent LOH/MSI alteration (31.25 %) in prostate carcinoma samples has been observed in 7q32.2 chromosomal region (D7S530, D7S2544, D7S2519 markers) which belongs to IR region, followed by 16q22.1 (26.60 %; D16S3025, D16S496 markers) belonging to NIR. The lowest frequency (6.80 %) of LOH/MSI was observed in 12q23.2 (D12S1041, D12S1727 markers) belonging to NIR. The total percentage of LOH/MSI was assessed for IR and NIR separately and revealed the value of 12.11 % for IR and 9.75 % for NIR (see Fig. 2).

Statistical analysis (Mann–Whitney *U* test) has not confirmed the significant difference in LOH/MSI frequency in IR versus NIR (*p* > 0.05). The frequency of genetic instability (LOH/MSI) in the studied prostate adenocarcinoma group was also assessed as FAL index, ranging from 0.00 to 0.93 (mean 0.10 ± 0.17). FAL indexes were evaluated only for those prostate adenocarcinoma samples, for which LOH/MSI was present at least in one microsatellite *locus*. The results are shown in Table 4.

**Table 4:** The assessed LOH/MSI frequency (FAL index) in prostate adenocarcinoma DNA samples for 16 studied microsatellite markers, mapped to: 1p, 3p, 7q, 9p, 12q, and 16q

Sample	D1S2137	D1S368	D3S3615	D3S1317	D3S3611	D7S2519	D7S2544	D7S530	D9S974	D9S1604	D11S4088	D11S1318	D12S1041	D12S1727	D16S496	D16S3025	FAL
23	I	NI	LOH	I	I	I	I	I	I	I	I	I	I	I	I	NI	0.07
33	LOH	I	I	I	I	I	NI	I	I	I	I	I	I	I	I	NI	0.07
44	I	I	NI	NI	NI	I	I	NI	LOH	NI	I	I	I	I	I	I	0.09
58	I	NI	LOH	I	I	I	I	I	I	NI	NI	I	NI	NI	I	I	0.09
102	I	I	I	I	I	I	NI	I	LOH	NI	NI	NI	LOH	NI	I	LOH	0.27
103	I	I	I	NI	I	I	I	NI	I	NI	I	MSI	NI	I	I	I	0.08
107	LOH	I	I	NI	I	I	I	I	I	I	LOH	LOH	NI	I	LOH	LOH	0.35
113	I	I	NI	I	LOH	I	LOH	I	I	I	I	I	I	I	LOH	LOH	0.26
119	LOH	I	I	NI	I	I	LOH	LOH	I	NI	I	I	I	I	NI	NI	0.25
120	NI	I	LOH	I	LOH	I	I	LOH	I	I	I	I	I	I	LOH	LOH	0.33
138	LOH	MSI	MSI	NI	MSI	MSI	I	MSI	MSI	LOH	LOH	MSI	MSI	LOH	LOH	LOH	0.93
142	I	NI	NI	NI	I	I	LOH	NI	NI	I	I	I	I	I	I	NI	0.10
149	I	NI	I	NI	I	LOH	LOH	LOH	NI	NI	I	I	NI	I	I	I	0.27
155	I	I	NI	I	I	I	NI	LOH	I	I	I	I	NI	I	I	I	0.08
162	I	NI	I	NI	I	LOH	NI	LOH	LOH	NI	I	I	NI	NI	I	I	0.30
166	MSI	NI	I	I	I	I	I	I	LOH	NI	NI	NI	NI	I	I	I	0.18
201	LOH	LOH	I	I	LOH	NI	I	NI	I	NI	I	I	I	I	NI	LOH	0.33
202	I	NI	NI	I	I	I	LOH	NI	I	I	I	NI	I	I	I	NI	0.09
216	NI	MSI	I	NI	I	I	I	I	I	I	I	NI	I	I	NI	I	0.08
225	I	LOH	NI	I	I	I	NI	I	I	I	I	I	NI	I	NI	I	0.08
231	LOH	I	I	NI	NI	LOH	I	I	LOH	NI	I	I	I	NI	LOH	LOH	0.42
238	MSI	LOH	NI	I	I	I	I	NI	LOH	NI	MSI	MSI	NI	I	I	I	0.42

*I* informative loci without LOH/MSI (heterozygote), *NI* non-informative allelotype (homozygote), *LOH* presence of LOH, *MSI* presence of MSI

### Correlation of LOH/MSI with clinicopathological parameters

**Fig. 3:**
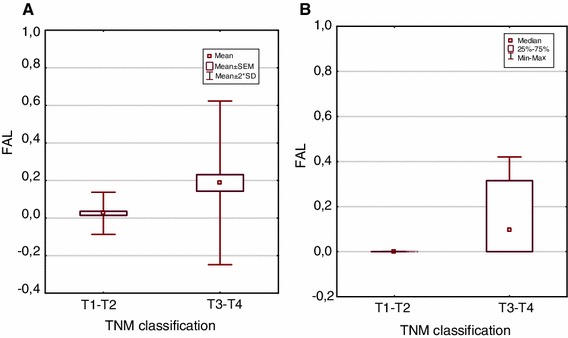
*Box-and-whisker plots*, representing the mean (**a**) and median (**b**) FAL index values of tumor size (T1–T2 and T3–T4 groups), according to TNM classification, in prostate adenocarcinoma group (Mann–Whitney test, *p* < 0.05)

Then, we assessed FAL index values of all (50) samples separately in relation to clinical features of patients: total PSA value (TPSA), total/free PSA value (T/FPSA), PSA density PSAD), prostate volume, and patient’s age at time of diagnosis as well as histopathological characteristics of tumors (according to TNM classification, and Gleason score). We observed that patients with FAL index ≥0.4 had diagnosed advanced tumors (in clinical and pathologic stage) according to TNM system (e.g., T3aNxM1, T3aNxMx or T3bNxMx). Especially, in case of one studied patient—with PSAT 1,489 ng/ml; PSAF 198.2; F/T PSA 0.13 and tumor stage T3bNxM1b—FAL index value of 0.93 was recognized (probe no 138, see Fig. 3). Mann–Whitney *U* test revealed statistically significant differences between FAL index levels and tumor size, according to TNM classification (*p* < 0.05; Fig. 3a, b).

There was no statistically significant correlation between FAL index levels and patients’ age, PSAD, TRUS image, DRE results (Mann–Whitney test, *p* > 0.05) as well as between FAL index values and Gleason score (Mann–Whitney test, *p* = 0.051). Moreover, no significant association between FAL index values and total PSA value, F/T PSA value, and prostate volume (Kruskal–Wallis test, *p* > 0.05) was observed.

## Discussion

In our study, we performed comprehensive allelotyping in chromosomal regions: 1p31.2, 3p21.3–25.3, 7q32.2, 9p21.3, 11p15.5, 12q23.2, and 16q22.1, which cover IRs as well as NIRs of human genome. We confirmed cumulative data of other authors who recognized high LOH frequency in prostate sporadic cancer in many regions [8, 11, 12]. Our results provided evidence for the presence of LOH/MSI alterations in prostate tumor cells with significant frequency, mainly in chromosomal regions: 7q32.2 and 16q22.1, followed by 1p31.2 and 3p21.3–25.3.

Pivotal role of 7q and 16q alteration in tumor development and progression has been claimed [8, 13–15], and association with tumor stage and aggressiveness has been emphasized [8, 15].

Interestingly, despite several studies evaluating LOH/MSI on 7q (7q31.1-32 region), no important gene *loci* in this region have been unequivocally identified [8, 14]. On the other hand, long arm of chromosome 7 possesses imprinted domain in 7q32-qter which including *MEST1*, *MESTIT1*, *COPG2IT1* *loci* and a group of four *carboxypeptidase A* (*CPA*) genes (*CPA1*, *CPA2*, *CPA4*, *CPA5*) [16], which are seem to be important (mainly *MEST*) in the initiation of breast and lung neoplasms [17]. Therefore, confirmed in our study high frequency of LOH/MSI in 7q32.2 may provide evidence that this region is also important in prostate carcinogenesis.

In our data, 16q 21.1 *locus* has been observed as next important region with significant (~30 %) frequency of LOH/MSI in the studied adenocarcinoma group. Our observation was confirmed by others [14]. According to the earlier studies-specific chromosomal region, 16q21-24 involved in large region (including 8p, 6q, and 18q distal and proximal to *DCC* gene) revealed AI in different types of human cancer including prostate [18, 19]. Our results focused on 16q22.1-22.3 seem to be innovative and possess a pivotal value.

Moreover, we did not confirm any correlations between clinicopathological parameters and frequency of LOH/MSI in any studied chromosomal regions, or in any separately examined *loci*. However, it should be stressed that in our study statistically significant correlation between total FAL index and advanced tumor stage has been documented. We assume that small numbers of tumors included in our study might influence the above-mentioned observation.

Very important aspect of our study seems to be associated with 1p31.2 *locus*, where statistically significant increase in LOH/MSI alteration has been observed (for marker D1S2137). According to earlier study focused on 1p region in prostate cancer susceptibility, only one *locus* known as CRAB has been recognized on 1p36 [20]. To our knowledge, two selected in our study markers for 1p31.2 region analysis (covering *ARHI* and *GADD45A* genes) have not been used in PCa study yet. Molecular downregulation of *ARHI* (including LOH) is proved to be linked with several cancer types in human, such as ovarian, breast, hepatocellular and thyroid cancer [10, 21, 22], but LOH observed by us in this *loci* in prostate carcinogenesis is innovative and should be continued as a new project.

In our study, LOH/MSI alteration was observed also in 3p21.3–25.3 region. We documented—as the first research team—frequent and statistically different LOH in *RASSF1A locus* (3p21.3). Our observation is important with regard to *RASSF1A* biological function as TSG in which inactivation has been implicated in wide variety of sporadic human cancers as a negative regulator of cell proliferation and apoptosis.

It should be stressed that statistically significant increase in AI in 9p21.3 region (including *CDKN2A/p16 locus*) confirmed in our study provides the evidence supporting the thesis about the presence of 9p21 gene cluster associated with increased tumorigenicity [23, 24].

As so far, there are only few studies concerning 11p and/or 12q, as these regions have not been recognized as a “hot spot” for AI in prostate carcinoma [25]. Our result conformed this observation but in our study focus on this regions LOH/MSI incidence was higher that assessed so far. In these chromosomal regions analyzed in our study, the following genes are located: *KCNQ1* (11p15.5), *CDKN1C* (11p15.5), and *SLC5A8* (12q23.2). In our opinion, especially the *CDKN1C* gene, negative regulator of cell proliferation alterated in many tumors may be important in prostate carcinogenesis [26, 27].

Based on some earlier study, comparing the frequencies of AI with the clinicopathological features and prognosis in prostate cancer, the obtained results are highly controversial [25, 28].

Reassuming, we identify major chromosomal regions (1p31.2, 7q32.2, 16q22.1 and 9p) in which LOH/MSI presence is connected with prostate cancer size (T in TNM staging). We suggest that total LOH/MSI alteration assessed as FAL index from biopsies may have clinical value for the molecular staging of prostate cancer in transrectal ultrasound (TRUS)-guided prostate biopsy material and seem to be promising.
